# Supplementary material for: Dynamic Variations in Endogenous Peptides in Chinese Human Milk Across Lactation and Geographical Regions
Source: Nutrients. 2025 Sep 30;17(19):3131. doi: 10.3390/nu17193131 (PMC12525668; doi:10.3390/nu17193131)
Supplement: Supplementary file 1 [file nutrients-17-03131-s001.zip › Supplementary Figures S1–S3.pdf]

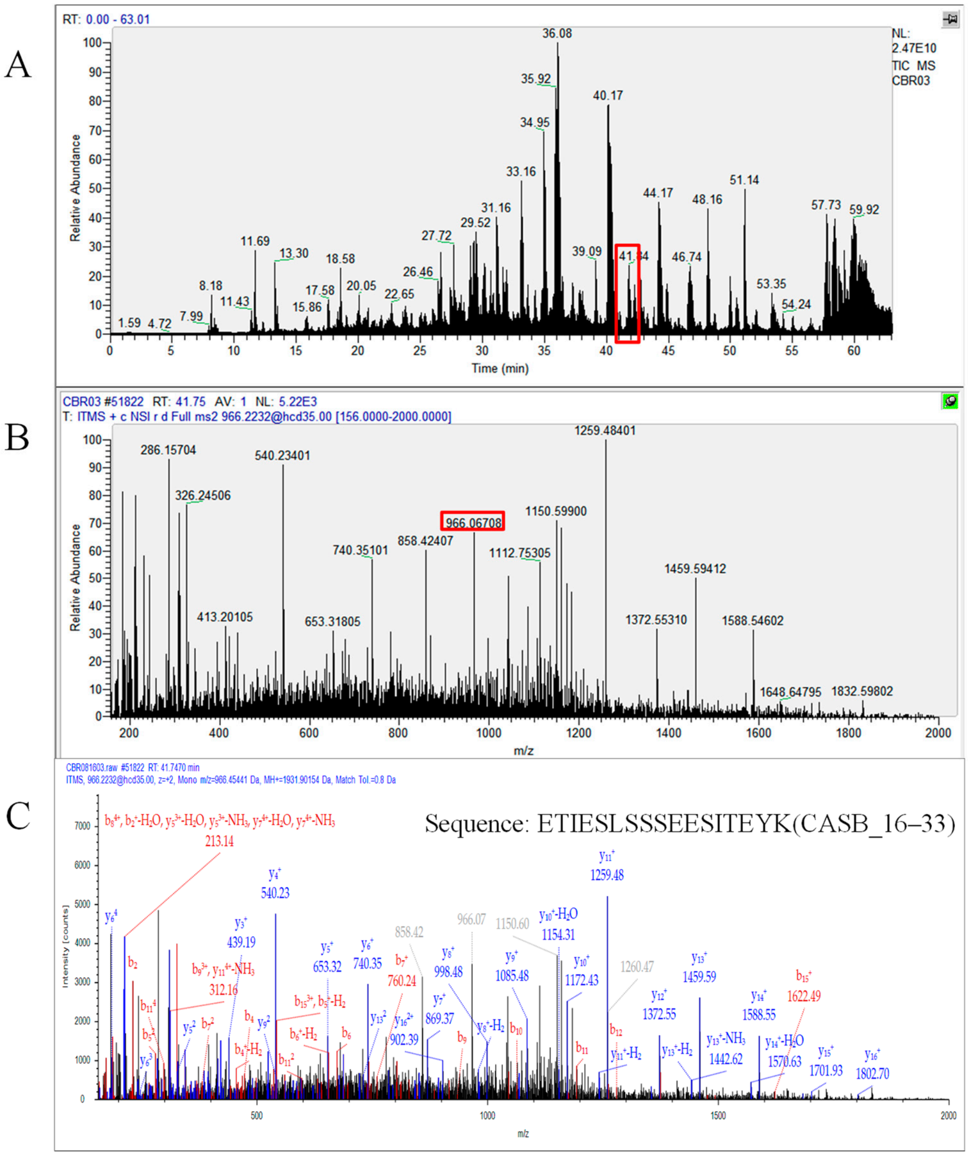

Figure S1. Exemplificative workflow for the identification of the peptide ETIESLSSSEESITEYK (CASB_16–33) in the 6-month breast milk sample. (A) HPLC-MS/MS total ion chromatogram (TIC) of endogenous peptides detected in 6-month milk. (B) MS 1 spectrum corresponding to the retention time (RT) of peptide CASB_16–33. (C) Sequence assignment of CASB_16–33 obtained from raw data analysis using Proteome Discoverer software (version 2.4, Thermo Scientific).

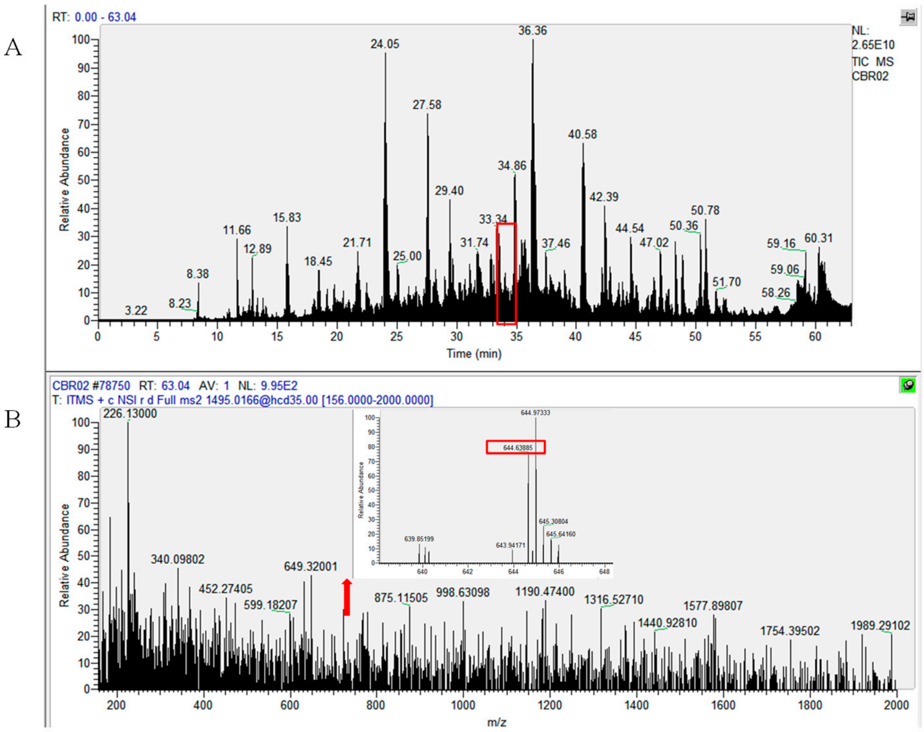

Figure S2. Exemplificative workflow for the identification of the peptide ETIESLSSSEESITEYK (CASB_16–33) in breast milk sample from Lanzhou. (A) HPLC-MS/MS total ion chromatogram (TIC) of endogenous peptides detected in milk from Lanzhou. (B) MS 1 spectrum corresponding to the retention time (RT) of peptide CASB_16–33. The inset in panel B shows a magnified view containing the m/z 644.63 signal.

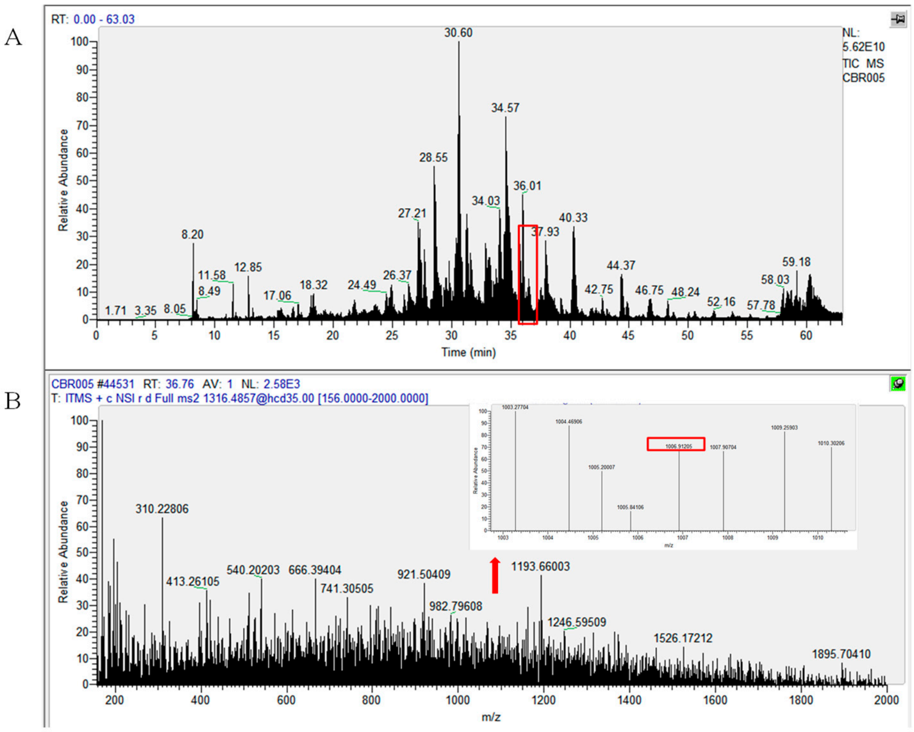

Figure S3. Exemplificative workflow for the identification of the peptide ETIESLSSSEESITEYK (CASB_16–33) in breast milk sample from Jinhua. (A) HPLC-MS/MS total ion chromatogram (TIC) of endogenous peptides detected in milk from Jinhua. (B) MS 1 spectrum corresponding to the retention time (RT) of peptide CASB_16–33. The inset in panel B shows a magnified view containing the m/z 1006.91 signal.
